# Factors affecting adherence in cardiovascular protective medications: An UMPIRE sub-study

**DOI:** 10.1186/1753-6561-9-S1-A35

**Published:** 2015-01-14

**Authors:** JM Peart, A Stanton

**Affiliations:** 1 Cardiovascular Department, Beaumont Hospital, Dublin 9, Ireland

## Background

Adherence to protective cardiovascular medications is of huge importance in the prevention of serious morbidity and mortality [1]. This study seeks to identify factors that influence adherence to the treatment of cardiovascular medications and to analyse the characteristics of the baseline non-adherence Irish cohort of the UMPIRE study. UMPIRE (“Use of a Multidrug Pill In Reducing cardiovascular Events”) was a prospective, randomised, open-label, blinded-endpoint (PROBE) clinical trial carried out in three locations in Europe (Ireland, The Netherlands and the UK) as well as India. The Irish cohort was a total of 333 participants (total trial 2,004 of which half in India). It had a key eligibility criterion of established cardiovascular disease (CVD) or an estimated 5-year CVD risk of ≥15%. The results were present at the American Heart Association 2012 Scientific Sessions.

## Methods

A literature review was carried out to identify evidence for factors affecting adherence in the treatment of hypertension. A database of patient profiles and health measurements was created and analysed using Excel and DataDesk. The unpaired Students t-tests and Chi-squared tests were used to test for differences between baseline adherent and non-adherent groups and the results were then compared with expectation from the literature review.

## Results

Several significant factors affecting adherence were suggested from the analysis of the Irish UMPIRE cohort. Younger age, a higher educational status and a lack of a Medical card were associated with non-adherence. There was a trend, consistent with the UMPIRE study, for higher systolic blood pressure, heart rate and LDL cholesterol levels in the non-adherent cohort as would be expected.

## Conclusions

Economic pressures appear to be a significant predictor of adherence which may account for the contrary findings to the literature review on age and educational status. In the Irish context, this might be attributed to younger middle income individuals having greater financial liabilities than their older peers on average.
